# Analysis of the transparent surface layer formed at the surfaces of arrested enamel caries lesions with tomographic imaging methods

**DOI:** 10.1016/j.jfscie.2023.100025

**Published:** 2023-06-20

**Authors:** Nai-Yuan N. Chang, Morgan Ng, Tina Dillas, Yi-Ching Ho, Yihua Zhu, Daniel Fried

**Affiliations:** a Department of Preventive and Restorative Dental Sciences, University of California, San Francisco, San Francisco, CA; b Department of Stomatology, Taipei Veterans General Hospital, Taipei City, Taiwan

**Keywords:** Optical coherence tomography, microcomputed tomography, lesion activity, arrested lesions, transparent surface layer

## Abstract

**Background:**

Optical coherence tomography (OCT) can acquire high-resolution images of lesion structure in vivo to assess lesion activity. The surface layers of arrested enamel caries lesions (ECLs) have a higher mineral content than the lesion body with a reduced porosity that inhibits the penetration of fluids into the lesion and appears with reduced reflectivity OCT. This study aimed to use microcomputed tomography (microCT) to measure the mineral content of the transparent surface layer (TSL) that appears on OCT images.

**Methods:**

OCT and microCT were used to measure the reflectivity and relative mineral density profiles of 20 bovine enamel samples that had simulated arrested ECLs, 44 extracted teeth with 17 proximal ECLs, 10 occlusal ECLs, and 17 lesions caused by fluorosis. TSL thickness (OCT), surface layer thickness (microCT), and the minimum and maximum densities of the TSLs were determined from matched OCT and microCT profiles.

**Results:**

For all samples, the TSL thickness measured with OCT was significantly less than the surface layer thickness measured with microCT (*P* < .05). Mean maximum mineral densities in the TSL were statistically similar to sound human enamel (*P* > .05) and significantly higher (*P* < .05) than the mean minimal mineral densities of the TSL.

**Conclusions:**

The maximum mineral densities of the TSL measurable with OCT approach those of sound enamel suggesting that almost complete remineralization has occurred in the TSLs. This study provides further confirmation that the presence of a TSL in OCT images is a key indicator of lesion arrest.

## Graphical abstract

**Figure:**
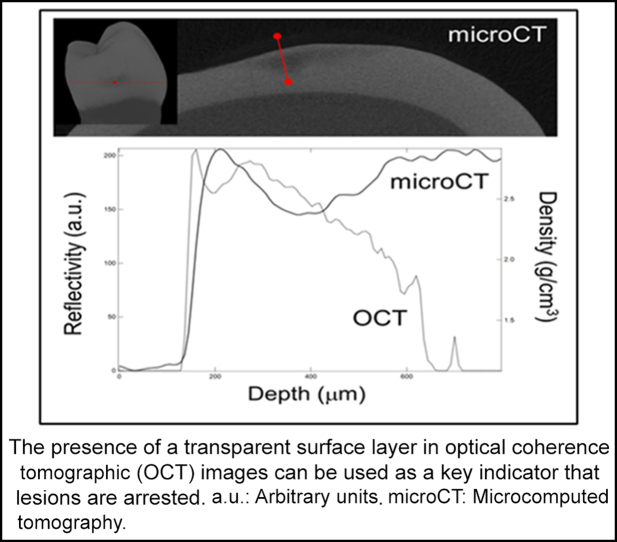

**Why Is This Important?**

Caries lesions can be arrested by the preferential deposition of minerals at the lesion surface that inhibits the diffusion of fluids into the porous lesion. Because arrested lesions do not need further intervention, assessing lesion activity is essential for caries management. Conventional methods of lesion activity assessment, such as visual and tactile methods, are unreliable. Studies have shown that optical coherence tomography (OCT) can measure lesion structure nondestructively in vivo. Arrested lesions have a surface layer of lower reflectivity than the lesion body in OCT images, called a transparent surface layer (TSL). This study aimed to compare OCT and microcomputed tomographic images of arrested lesions to determine how high the mineral content needed to be in the TSL to appear transparent in OCT images. This study shows that the mineral density in the TSL approaches that of sound enamel and that almost complete remineralization has occurred in this zone. This strongly supports the hypothesis that a TSL near the lesion surface in OCT images is a key indicator of lesion arrest.

## Introduction

An accurate assessment of lesion activity, depth, and severity is necessary for effective caries management. Caries lesions are arrested by the preferential deposition of minerals at the lesion surface, inhibiting the diffusion of fluids into the porous lesion.^1^, ^2^, ^3^ Because arrested lesions do not need further intervention, assessing lesion activity is essential for clinical diagnosis. Many lesions have been arrested or do not require further intervention. It is difficult to identify active lesions with diagnostic methods. Accepted reference standards for lesion assessment, such as transverse microradiography and polarized light microscopy, require destruction and sectioning of the tooth for examination in cross-section.^4^ Modern microcomputed tomographic (microCT) systems now have sufficient resolution to resolve the internal structure of caries lesions, including the highly mineralized surface layer (SL) on intact teeth; however, such systems cannot be used in vivo.^5^^,^^6^ Several in vitro studies using models of demineralization and remineralization have shown that a highly mineralized surface layer is formed at lesion surfaces after exposure to a remineralization solution and that the SL appears with reduced reflectivity compared with the underlying lesion body (LB) in optical coherence tomographic (OCT) images. The SL appears more transparent and can be referred to as the transparent surface layer (TSL).^7^, ^8^, ^9^, ^10^ TSLs have been measured using OCT on extracted teeth with proximal and occlusal enamel caries lesions (ECLs) and in vivo on suspected arrested lesions on coronal and root surfaces.^11^, ^12^, ^13^, ^14^, ^15^ OCT imaging studies have shown that many lesions caused by fluorosis also have distinct TSLs at lesion surfaces, and they appear similar in structure to arrested ECLs.^16^^,^^17^ OCT is the only tomographic method with the sufficient resolution that can be used to assess lesion structure and activity and measure the thickness of the TSL in vivo.

The role of the TSL in arresting lesions and reducing their permeability to fluids has been confirmed by various approaches for monitoring lesion dehydration dynamics. Arrested lesions are less permeable to water because of the highly mineralized SL; therefore changes in the rate of water loss can be related to changes in lesion structure and porosity. Changes in fluorescence loss,^18^, ^19^, ^20^ thermal emission,^15^^,^^21^^,^^22^ and short wavelength infrared reflectance^23^, ^24^, ^25^, ^26^, ^27^ during lesion dehydration have been evaluated. Loss of that water from pores near the lesion surface due to evaporation produces a marked increase in light scattering, increasing the reflectivity and loss in fluorescence intensity. In addition, the evaporation of water is highly endothermic and produces a measurable drop in temperature and thermal emission from the lesion surface. Short wavelength infrared and thermal imaging methods confirm that a measurable TSL in OCT images correlates with decreased lesion permeability, indicating lesion arrest.^23^^,^^25^^,^^26^ There is a negative association between the SL thickness and lesion permeability; a small increase in the SL thickness of less than 20 μm can lead to a marked decrease in permeability.^28^ In addition, in a closely related study, the SL was removed from arrested lesions, producing a corresponding rise in the permeability, which further confirms the role of the SL in arresting lesions.^29^

It is unclear how high the mineral content has to be in the highly mineralized SL for it to appear with reduced reflectivity in OCT images. This study aimed to use microCT to determine the relative mineral content of the TSLs identified in OCT images of simulated ECLs, arrested ECLs on the proximal and occlusal surfaces of extracted teeth and lesions caused by fluorosis.

## Methods

### Simulated arrested ECLs (bovine blocks)

Twenty blocks were prepared for this study from extracted bovine incisors from a slaughterhouse. Flattened bovine enamel blocks are a suitable substitute for human enamel in the study of caries and yield more uniform lesions on demineralization.^30^ Each block was approximately 10 through 14 mm long, 3 mm wide, and 2 mm thick. Each sample was partitioned into 5 windows by etching small incisions 2.5 mm apart across each block using a carbon dioxide laser. Lesions were produced approximately 80 through 100 μm deep by exposing the center 3 windows to pH cycling for 6 days.^31^ The central lesion window was subsequently exposed to a neutral remineralization regimen for 12 days to remineralize that window and produce a simulated ECL. A thin layer of acid-resistant varnish was applied to all sides except the top surfaces of the enamel blocks before exposure to the demineralization and remineralization solutions. Windows not undergoing treatment were covered with acid resistance varnish when immersed in the demineralization and remineralization solutions. The initial lesions were produced by pH cycling in 40-mL aliquots of the demineralization solution for 7 hours, followed by the neutral remineralization solution without fluoride for 17 hours daily for 6 days. The demineralization solution was maintained at 37 °C at pH 4.8, and it comprised 2.0 mmol/L calcium, 2.0 mmol/L phosphate, and 75 mmol/L acetate. The remineralization solution comprised 1.5 mmol/L calcium, 0.9 mmol/L phosphate, 150 mmol/L potassium chloride, and 20 mmol/L HEPES buffer maintained at pH 7.0 and 37 °C. After lesions were produced in the central 3 windows, the central window was exposed only to the remineralization solution continuously for 12 days, respectively. Two parts per million of fluoride were added to the neutral remineralization solution described above to enhance remineralization. Simulated lesions have been assessed previously using OCT.^8^, ^9^, ^10^^,^^12^^,^^32^^,^^33^

### Extracted teeth with ECLs on proximal and occlusal surfaces and fluorosis

Permanent teeth (N = 43) extracted from oral surgeons in the San Francisco, California, area were collected, cleaned, sterilized with gamma radiation, and stored in a 0.1% thymol solution. Permanent teeth were likely extracted for orthodontic reasons and consisted of premolars and molars. Teeth were selected with ECLs on proximal (n = 17) and occlusal surfaces (n = 10) and with fluorosis (n = 17).

### Optical coherence tomography

An IVS-2000-HR-C OCT system (Santec) that uses a swept laser source and a handpiece with a microelectromechanical scanning mirror and the imaging optics was used to scan the samples. The body of the handpiece was 7 × 8 cm with an imaging tip that was 4 cm long and 1.5 cm across. Complete tomographic images with a volume of 5 × 5 × 5 mm were captured in approximately 3 seconds at a wavelength of 1,312 nm with a bandwidth of 173 nm and a measured air resolution of 8.8 μm (3 dB). Measured lesion depths (LDs) and TSLs were divided by 1.6, the refractive index of enamel. The lateral resolution is 30 μm (1 × 10^2^) with a measured imaging depth of 5 mm and depth resolution of 5 μm in air.

**Figure 1:**
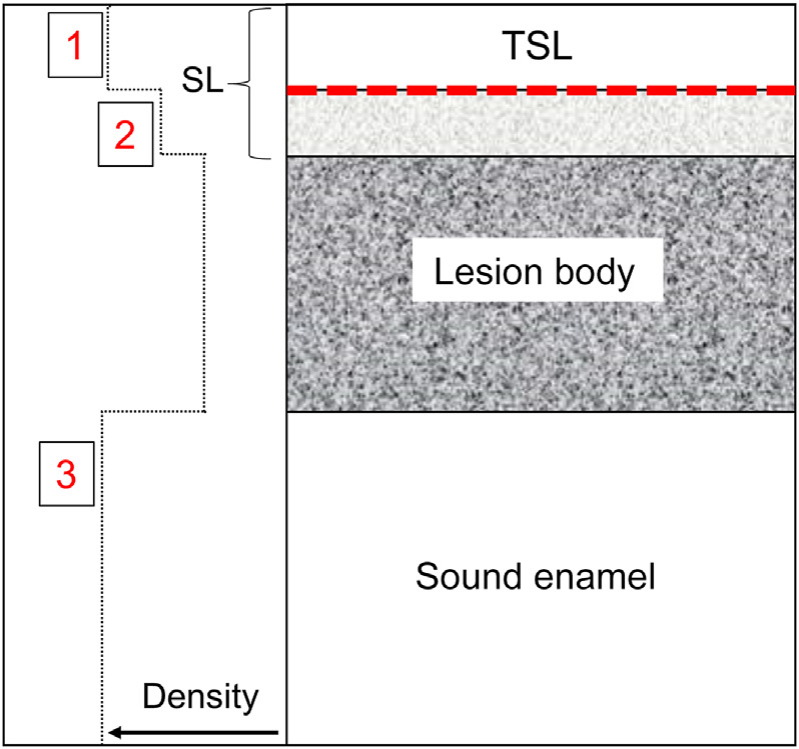
Diagram of a typical enamel caries lesion showing how the transparent surface layer (TSL) and surface layer (SL) were defined. The TSL is the layer that appears more transparent in optical coherence tomographic images. The SL is the outer layer with a lower density than the body of the lesion. A profile of the mineral density is represented by the dotted black line on the left. The density increases from right to left. This study measured the thickness of the TSL and the SL. In addition, the densities were reported at 3 positions (1-3), the maximum density in the SL (position 1), the minimum density of the TSL at the position matching the full thickness of the TSL as indicated by the dashed red line (position 2), and the density of the underlying sound enamel (position 3).

Image analysis and lesion structural measurements were performed using Dragonfly (ORS). OCT images were filtered using a 5-pixel median filter to despeckle the images, and an image mask with window-level thresholding was applied to designate the lesion area. For quantitative measurements of the TSL thickness, a single location at the surface of each sample was selected, and the corresponding intensity profile of intensity vs depth was extracted orthogonal to the sample surface (S) with a diameter of 100 pixels. The TSL thickness was measured as the distance between the center of the first 2 peaks representing S and the beginning of the LB. A diagram showing how the TSL is defined along with the SL measured with microCT is shown in Figure 1.

### Microcomputed tomography

Samples were imaged using microCT with a 10-μm resolution. A Scanco MicroCT 50 (Scanco USA) located at the University of California San Francisco Bone Imaging Core Facility was used to acquire the images.

**Figure 2:**
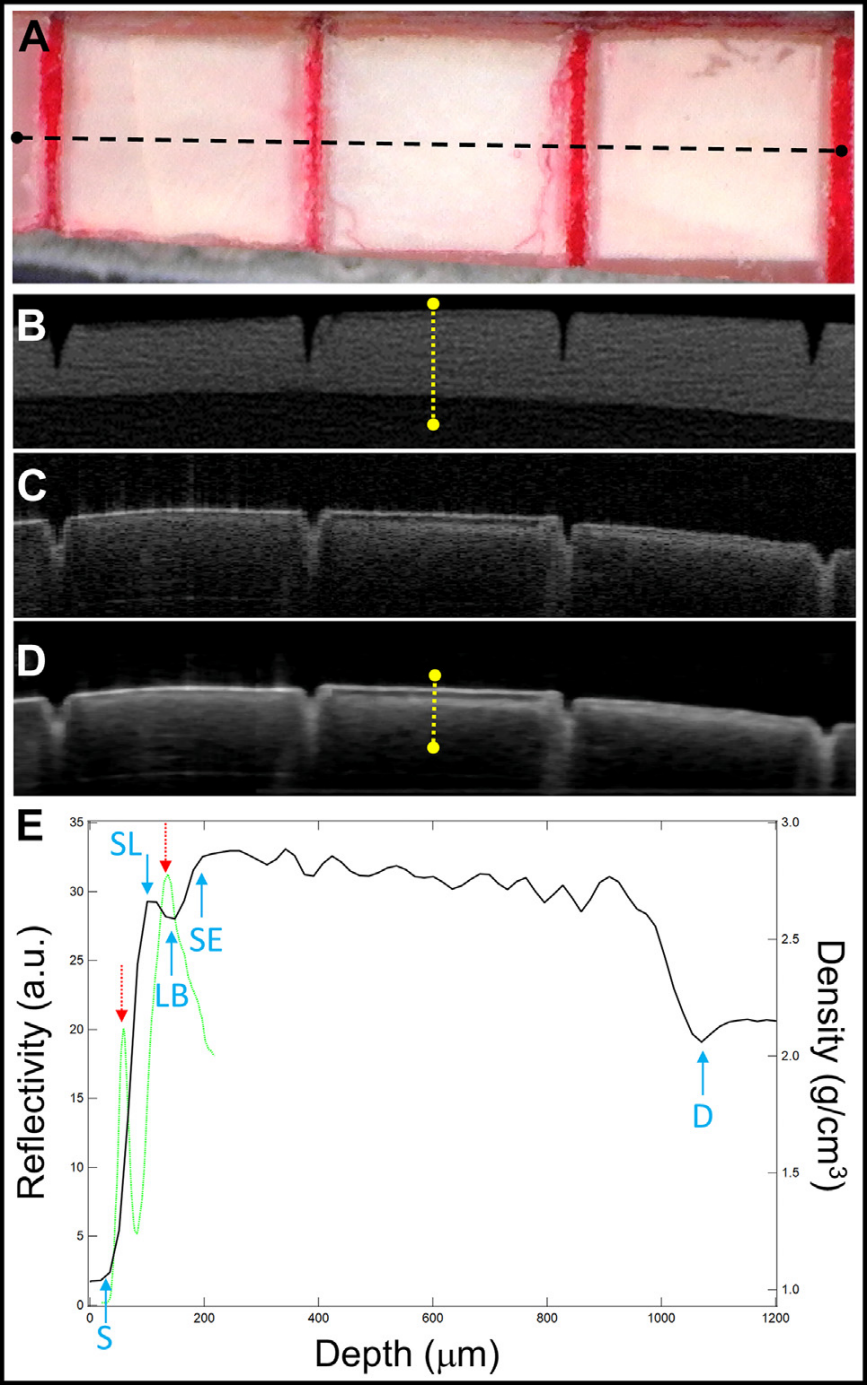
**A.** Color image of a bovine enamel block 2 × 10 mm, showing the center 3 windows. The optical coherence tomographic (OCT) and microcomputed tomographic (microCT) cross-sectional images shown were extracted from the position of the dashed black line. Matching extracted microCT images (**B)**, raw images **(C)**, and processed OCT **(D)** cross-sectional images are shown. The yellow dotted lines show the position of the extracted line profiles in panel **E**. Line profiles from OCT (dotted green line) of reflectivity vs depth and microCT (solid black line) of density vs depth are shown. The transparent surface layer is defined as the distance between the 2 dotted red arrows representing the reflection from the surface and the beginning of the lesion body (LB) on the OCT profile. The position of the sample surface (S), the surface layer (SL), LB, beginning of sound enamel (SE) and dentin (D) are indicated by the solid blue arrows on the microCT line profile. a.u.: Arbitrary units.

Acquisition parameters used for the microCT images were as follows: 90 kVP, 200 uA, a field of view of 18 × 20, 10-μm voxel size, 500 milliseconds integration time, and an aluminum 0.5 mm filter. Voxel intensities were converted into densities in g/cm^3^ with a custom MATLAB script that converts the image to density using water, sound enamel (SE), and sound dentin (D) on each sample with known densities (water = 1.0 g/cm^3^; SE = 3.0 g/cm^3^; D = 2.1 g/cm^3^) and generated a linear fit to complete the conversion. The converted density values are relative and not absolute intensity values because SE and dentin density vary slightly from sample to sample and within each tooth. However, the intensity vs. density plots for the 3 tissue types on each sample was highly linear, with Pearson correlation coefficients (*r*^*2*^) exceeding 0.99. The converted microCT image with every voxel displayed in g/cm^3^ was used for quantitative measurements. Matching locations were selected from the OCT and microCT images for each lesion and a corresponding intensity profile of density vs depth was extracted from the microCT image orthogonal to S with a diameter of 100 μm. The SL thickness and lesion depth (LD) were measured on the profile. The SL thickness was measured as the distance between the rise in mineral content at S to the end of the first peak representing the SL. These 2 positions are shown by the 2 solid blue arrows (labeled S and LB) in Figure 2E.

In addition, 3 mineral density values were extracted from the microCT profile, as indicated in Figure 1. The first corresponds to the maximum mineral density of the TSL or the peak density. The second corresponds to the position matching the depth or thickness of the TSL measured from the matching OCT line profile (dashed red line in Figure 1). This represents the highest reflectivity of the TSL and the minimum density, in which the reflectivity is similar to the underlying LB. The third value is the density of the underlying SE.

### Statistical analysis

Repeated measures analysis of variance with Tukey-Kramer post hoc multiple comparison tests were used to compare the sound and minimum and maximum TSL mineral densities measured with microCT. Paired 2-tailed *t* tests were used to compare the TSL thickness measured with OCT with the SL thickness measured with microCT. The TSL and SL thickness data were normally distributed according to the D’Agostino-Pearson test. The sound density values were normally distributed for all the lesion types. However, not all the minimum and maximum TSL densities were normally distributed. The Pearson correlation coefficient is reported for linear correlations between TSL and SL measurements. All statistical analyses were performed using Prism (GraphPad Software) with a significance level of 0.05.

## Results

The mean (SD) thickness of the TSL measured using OCT, and the thickness of the SL of higher mineral content measured with microCT for the 4 lesion types are tabulated in the Table. In addition, the mean (SD) for the maximum and minimum densities of the TSL and SE are listed.

**Table:** TSL∗ and surface layer thickness measurements and minimum and maximum densities of the TSL and the underlying sound enamel for the 4 lesion types**.**†

Lesion Type	Bovine	Proximal	Occlusal	Fluorosis
Sample size (n)	27	17	10	16
Thickness (μm), mean (SD)				
Optical coherence tomographic TSL	24 (8.6)	81 (27)	66 (28)	86 (32)
Microcomputed tomographic surface layer	69 (13)‡	160 (51)‡	132 (35)§	123 (39)‡
Density (g/cm^3^), mean (SD)				
TSL (minimum)	2.58 (0.24)	2.78 (0.17)^a^	2.38 (0.23)^a^	2.78 (0.24)^a^
TSL (% sound enamel)	84.0 (9.0)	79.0 (7.2)	92.0 (5.8)	92.0 (8.0)
SL (maximum)	NA#	2.95 (0.10)^b^	2.87 (0.16)^b^	3.05 (0.13)^b^
SL (% sound enamel)	NA	98.0 (3.5)	96.0 (5.4)	101.0 (4.3)
Sound, mean (SD)	NA	3.01 (0.02)^b^	3.00 (0.03)^b^	3.01 (0.01)^b^

∗ TSL: Transparent surface layer.

† For the multiple comparisons of the densities, columns with the same letter are statistically similar (*P* > .05), whereas columns with a different letter are significantly different (*P* < .001).

‡ *P* < .001.

§ *P* < .0001 for the *t* tests indicating the microcomputed tomographic surface layer is significantly larger.

# NA: Not applicable.

### Bovine simulated ECLs

Among the 40 simulated ECLs, 27 samples produced TSLs measurable by OCT. The TSL thickness ranged from 10 through 41 μm with a mean (SD) of 24.0 (8.6) μm. The SL thickness of these samples ranged from 46 through 100 μm with a mean (SD) thickness of 69 (13) μm. The minimum mean (SD) density of the TSL was 2.58 (0.24) g/cm^3^, 84% (9%) that of the density of sound bovine enamel. The correlation of TSL and SL thickness was significant and weakly positive (*r*^*2*^ = 0.11; *P* < .05).

Color, microCT, and OCT images of 1 of the bovine samples are shown in Figure 2. Only the central window with the lesion produced by pH cycling for 6 days and subsequently exposed for 12 days to the neutral remineralization solution was used for this study because this window produced the largest TSLs and the most consistent remineralization. The microCT image (Figure 2B) shows a thin dark line below the surface, indicating the lower mineral content LB. The thin whiter line above that layer is the SL of higher mineral content, and it appears similar in density to that of the SE. The OCT images in Figures 2C and D show a thin white line at the surface of high reflectivity, indicating the specular reflection from the surface. The TSL is the dark gap of reduced reflectivity between the surface reflection, and the beginning of the LB that appears between 2 peaks (2 dotted red arrows) in the extracted line profile of reflectivity vs depth is shown in Figure 2E. The underlying LB has a higher reflectivity than the surrounding SE. The TSL and SL appear with opposite intensity in the OCT and microCT images, darker in the OCT image and lighter in the microCT image. Features in the matching line profile of density vs depth in Figure 2E extracted from the microCT image are indicated by the solid blue arrows showing S, SL, LB, SE, and D. The position of the initial rise in density marks the S in the microCT profiles, whereas the center of the first peak marks the position of the surface in OCT images.

### Proximal ECLs

For the 17 proximal ECLs, the mean (SD) LD measured by microCT was 546 (23) μm. The mean (SD) TSL thickness was 81 (27) μm, whereas the mean (SD) SL thickness was 160 (51) μm. The TSL and SL thickness correlation was positive but not significant (*r* = 0.16; *P* > .05).

The minimum density of the TSL was significantly less (*P* < .001) than that of the SE and the maximum density of the TSL; there was no significant difference between the maximum TSL density and the SE density (*P* > .05).

**Figure 3:**
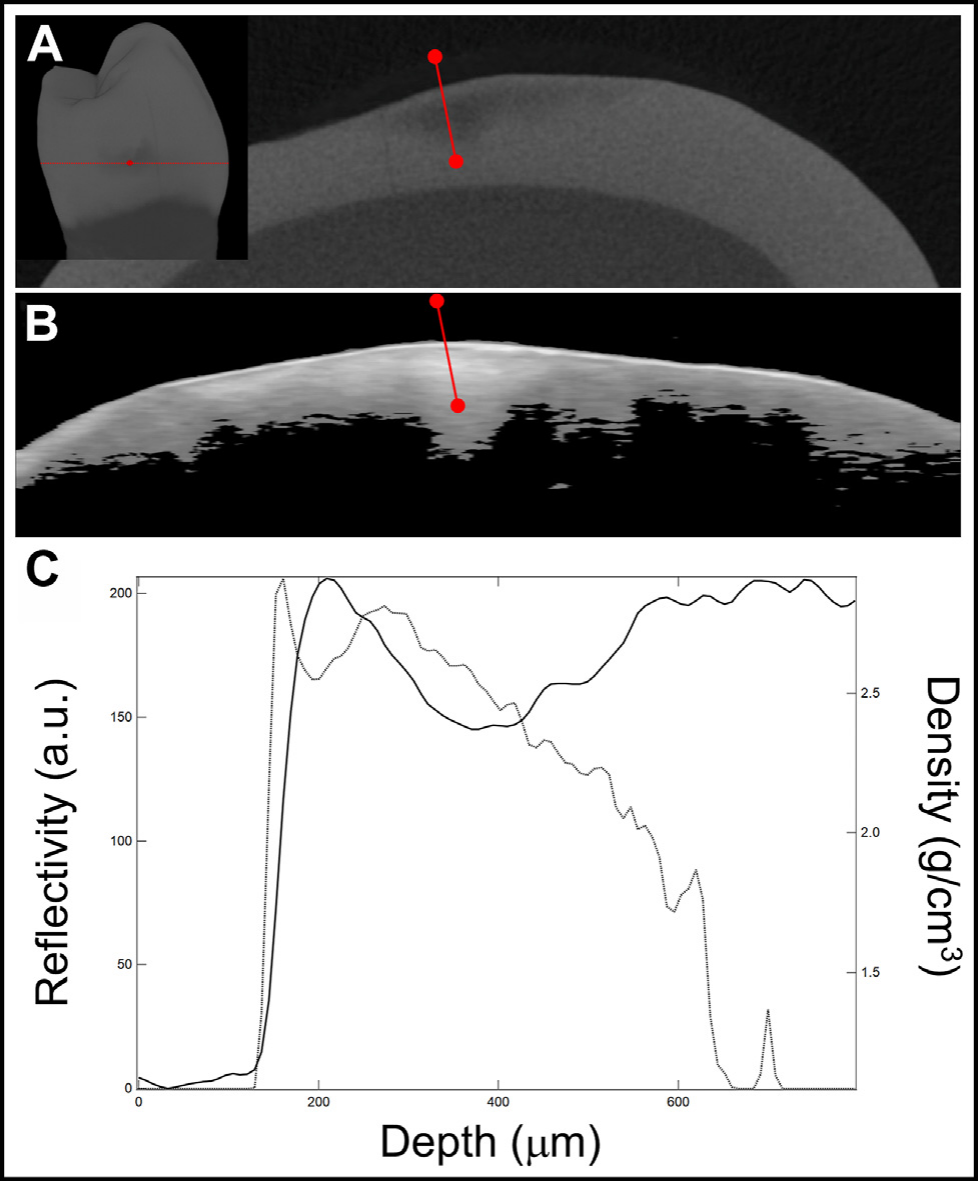
Microcomputed tomographic (microCT) surface rendering of an extracted tooth with a lesion on the proximal surface showing the position at which the microCT **(A)** and processed optical coherence tomographic **(B)** cross-sectional images shown were extracted from the position of the dotted red line. The solid red lines show the position of the extracted line profiles shown in **(C)**. **C.** Line profiles from optical coherence tomography (dotted line) of reflectivity vs depth and microCT (solid line) of density vs depth are shown. a.u.: Arbitrary units.

OCT and microCT images of one of the proximal ECLs are shown in Figure 3. The TSL and SL thicknesses are much larger for naturally formed arrested lesions on extracted teeth than for the simulated arrested lesions formed on bovine enamel.

### Occlusal ECLs

For the 10 occlusal ECLs, the mean (SD) LD measured by microCT was 598 (22) μm. The mean (SD) TSL thickness measured by OCT was 66 (28) μm, whereas the mean (SD) SL thickness was 132 (35) μm. The correlation of TSL and SL thickness was positive but was not significant (*r* = 0.31; *P* > .05).

The minimum density of the TSL was significantly less than the maximum density within the TSL and within the SE, respectively (*P* < .001). There was no significant difference between the maximum TSL/SL and SE densities (*P* > .05).

**Figure 4:**
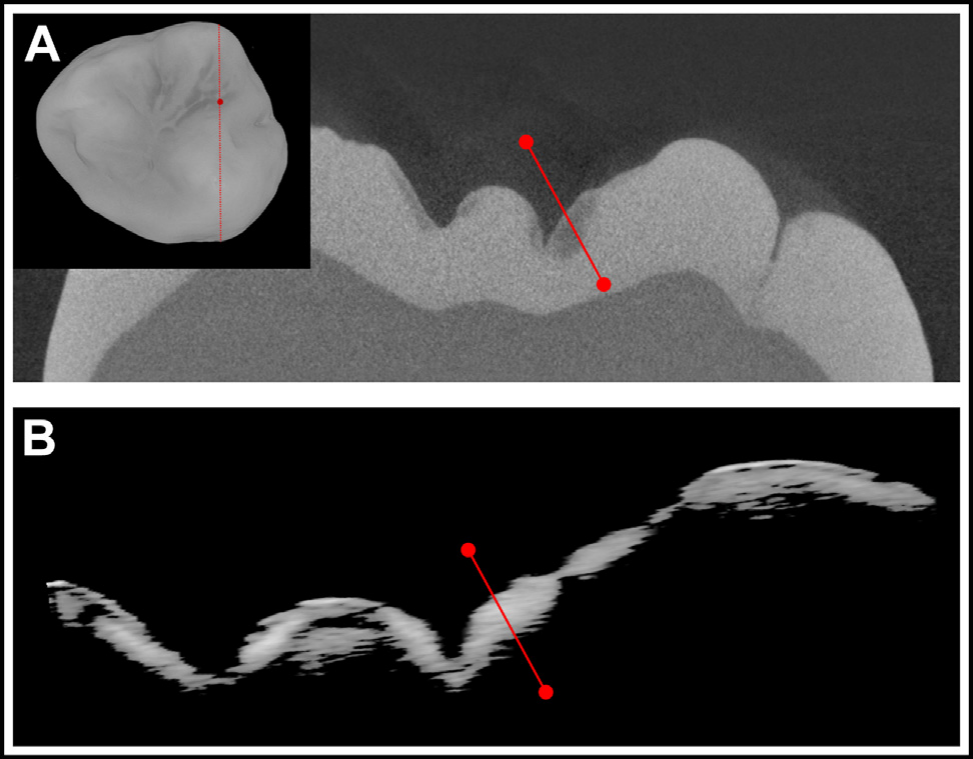
Microcomputed tomographic surface rendering of the occlusal surface of a tooth with occlusal lesions showing the position at which the microcomputed tomographic **(A)** and processed optical coherence tomographic **(B)** cross-sectional images shown were extracted from the position of the dotted red line. The solid red lines show the position that was chosen for analysis.

OCT and microCT images of 1 of the occlusal lesions are shown in Figure 4. Although the OCT and microCT images were coregistered, there is some distortion between the 2 cross-sectional images because of differences in the physical and optical path length that OCT measures. Such differences are more apparent on the highly convoluted occlusal surface.

### Fluorosis

**Figure 5:**
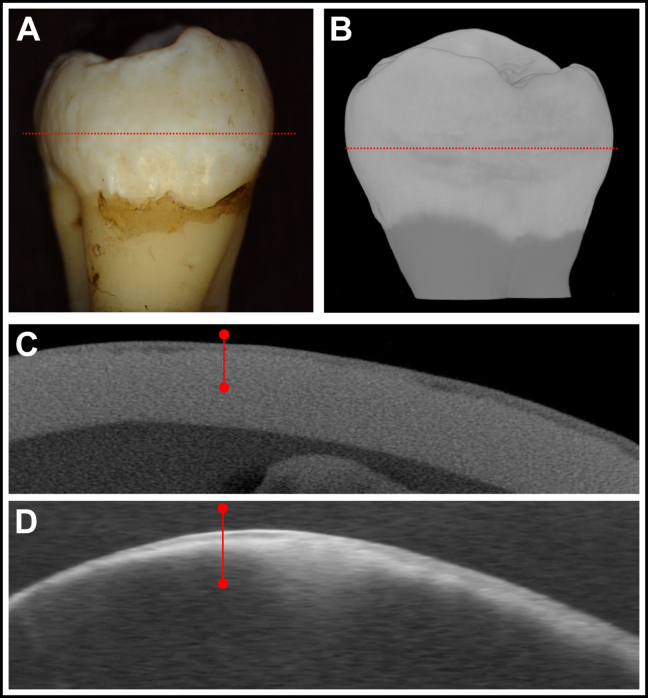
Color image **(A)** and microcomputed tomographic **(B)** surface rendering of the buccal surface of a tooth with mild fluorosis showing the position at which the microcomputed tomographic **(C)** and processed optical coherence tomographic **(D)** cross-sectional images shown were extracted from the position of the dotted red line. The solid red lines show the position that was chosen for analysis.

In the fluorosis group, 16 of 17 samples had TSLs measurable by OCT. The TSL thickness ranged from 40 through 143 μm with a mean (SD) of 86 (32) μm. The SL thickness ranged from 84 through 227 μm with a mean (SD) of 123 (39) μm. The correlation of TSL and SL thickness was significant and positive (*r*^*2*^ = 0.42; *P* < .05). The minimum density of the TSL was significantly less than the maximum density within the TSL and SE, respectively (*P* < .001). There was no significant difference between the maximum TSL/SL and SE densities (*P* > .05). OCT and microCT images of 1 of the developmental defects because of fluorosis are shown in Figure 5. The distinct SLs are visible in the OCT and microCT images.

## Discussion

Lesion activity assessment is necessary for lesion diagnosis because it can be used to determine whether treatment is necessary and whether intervention is effective. Mineral deposition in the pores near the surface of the lesion during remineralization increases the mineral content near the surface and forms an SL. This highly mineralized SL reduces the permeability of fluids preventing further demineralization and remineralization, arresting lesion progression and repair. Established methods for measuring lesion structure, such as transverse microradiography and polarized light microscopy, require the destruction of the tooth and are not suitable for use in vivo, whereas it has been shown that OCT can be used to measure TSL thickness for ESLs on proximal and occlusal surfaces, developmental defects (fluorosis) and even root caries nondestructively in vivo.^13^^,^^14^^,^^34^^,^^35^ In this study, we have shown that the maximum mineral density in the TSLs measured with OCT is statistically similar to that of SE. No accepted reference standard can be used clinically to assess lesion activity. This study provides further confirmation that the presence of a TSL in OCT images is a key indicator of lesion arrest.

This study also shows the mineral content or porosity reduction necessary to render the SL transparent in OCT images. Almost all active and arrested caries lesions have an SL with a higher mineral content or density than the LB.^1^ In the case of active lesions, the mineral content is not high enough to sufficiently inhibit further lesion progression. In addition to having SLs that are not visible in OCT images, active lesions also have different dehydration dynamics than arrested lesions because of the higher porosity of the outer SL. Time-resolved reflectivity measurements during the drying of lesions with forced air at wavelengths that coincide with water absorption bands such as 1,450 and 1,950 nm show a significant delay between the time the air is turned on and the onset of the increase in reflectivity.^27^ This delay occurs because the water in the pores of the outer SL absorb the incident light; when the water in those pores is lost, the reflectivity rises rapidly. Such a delay is not observed in arrested lesions because there are few pores in the highly mineralized TSL. In addition, with active lesions, once the reflectivity increases, it rises rapidly to reach the maximum reflectivity. In contrast, the reflectivity increase for arrested lesions is much slower and takes longer to reach the maximum reflectivity.^23^^,^^25^^,^^27^^,^^28^^,^^36^^,^^37^ Such differences in the rate of water loss are due to the different lesion structures; the TSL on the arrested lesions greatly inhibits the loss of water from deeper pores in the underlying LB.

For all the lesion types investigated, the SL measured with microCT was significantly larger than the thickness of the TSL measured with OCT. Measurements of the mineral loss of ECLs compared with optical attenuation at 1,300 nm indicated that optical attenuation continually increased until reaching mineral loss values approaching 15%; after that threshold, there was no further increase in attenuation or light scattering.^38^ Light scattering is caused by pores in the lesion, and with further mineral loss, the pores begin to merge to form larger pores, which does not lead to increased scattering and attenuation. Because OCT measures the increased reflectivity or light scattering from lesion areas at 1,300 nm, the optical attenuation should mirror the reflectivity measured with OCT. Therefore, areas of the SL with mineral loss values greater than 10% through 15% are expected to all appear with maximum reflectivity in OCT images and have similar reflectivity to the LB. In this study, the maximum mineral loss (minimum density) in the TSLs for ECLs ranged from 8% through 16%, with SDs that ranged from 6% through 9%. Therefore, the values measured in this study are consistent with the maximum 10% through 15% mineral loss values previously reported for maximum attenuation or light scattering at 1,310 nm.^38^ For active lesions, when the mineral loss in the outer SL exceeds 10% through 15%, the reflectivity is expected to be similar to that of the LB and no TSL is expected to be visible in OCT images.

The correlation between the TSL and SL thicknesses was low. There was only a significant correlation (*P* < .05) for the bovine lesions and the lesions because of fluorosis, and the correlation was weak to moderate. The bovine and fluorosis samples are shallower lesions with thinner SLs than the proximal and occlusal ECL, which may explain the higher correlation.

We anticipated that there would be major differences in the magnitude of the respective mean thickness of the TSLs formed on fluorosis and proximal ECLs lesions because of the different formation mechanisms. However, the mean TSL thickness was similar for both lesion types. The mean density of the TSL/SL for the fluorosis was higher than for the proximal ECLs and even exceeded the mean density of the surrounding SE. This was unsurprising because such TSLs are expected to have a high fluorapatite content.

## Conclusions

Our study shows that the mineral density of the outer SL in ECLs that appears transparent in OCT images, the TSL approaches that of SE and is significantly thinner than the entire outer SL of higher mineral content. OCT is the only tomographic imaging method that can be used in vivo to assess lesion structure and activity and measure the thickness of the TSL.

There are no approved dental OCT systems available for clinical use. Use in other medical areas, particularly in ophthalmology, is widespread, and there are no technological challenges in developing systems for dentistry. Commercial OCT systems are readily available that can be easily adapted for use in clinical studies of lesion activity.

## Disclosure

None of the authors reported any disclosures.
